# The Progress of Hard Carbon as an Anode Material in Sodium-Ion Batteries

**DOI:** 10.3390/molecules28073134

**Published:** 2023-03-31

**Authors:** Suchong Tan, Han Yang, Zhen Zhang, Xiangyu Xu, Yuanyuan Xu, Jian Zhou, Xinchi Zhou, Zhengdao Pan, Xingyou Rao, Yudong Gu, Zhoulu Wang, Yutong Wu, Xiang Liu, Yi Zhang

**Affiliations:** 1 School of Energy Sciences and Engineering, Nanjing Tech University, Nanjing 211816, China; 2 Jiangsu Svace Intelligent Technology Co., Ltd., Nanjing 210023, China

**Keywords:** sodium-ion battery, electrochemical performance, sodium-ion storage, hard carbon anode

## Abstract

When compared to expensive lithium metal, the metal sodium resources on Earth are abundant and evenly distributed. Therefore, low-cost sodium-ion batteries are expected to replace lithium-ion batteries and become the most likely energy storage system for large-scale applications. Among the many anode materials for sodium-ion batteries, hard carbon has obvious advantages and great commercial potential. In this review, the adsorption behavior of sodium ions at the active sites on the surface of hard carbon, the process of entering the graphite lamellar, and their sequence in the discharge process are analyzed. The controversial storage mechanism of sodium ions is discussed, and four storage mechanisms for sodium ions are summarized. Not only is the storage mechanism of sodium ions (in hard carbon) analyzed in depth, but also the relationships between their morphology and structure regulation and between heteroatom doping and electrolyte optimization are further discussed, as well as the electrochemical performance of hard carbon anodes in sodium-ion batteries. It is expected that the sodium-ion batteries with hard carbon anodes will have excellent electrochemical performance, and lower costs will be required for large-scale energy storage systems.

## 1. Introduction

Lithium-ion batteries are the main energy storage systems for mobile electronic devices such as electric vehicles, mobile phones, and laptops [1,2]. The lithium-ion batteries are the most mature energy storage devices at present, but their high cost and poor safety stagnate their commercial application [3,4,5]. Researchers are committed to finding products with a capacity and service life comparable to lithium-ion batteries, with lower costs and better safety performance, to satisfy the energy storage requirements of large-scale power grids in the country [6,7,8,9,10,11,12,13,14,15,16]. After long-term efforts, scientists finally found that sodium-ion batteries are the most promising alternatives to lithium-ion batteries [17,18,19,20,21,22,23]. Through investigation, it was found that the crustal abundance of sodium metal (2.74%) is more than 420 times that of lithium metal (0.0065%), which is rich in resources and evenly distributed worldwide [24,25,26]. Moreover, the two elements are of the same main group and have similar physical and chemical properties and similar electrochemical behaviors, and many technologies applied to lithium-ion batteries can also be applied to sodium-ion batteries [27,28,29,30,31,32,33]. Sodium-ion batteries have attracted wide attention as a substitute for lithium-ion batteries [34,35,36,37,38]. Anode materials play an important role in facilitating sodium-ion batteries with outstanding electrochemical performance. The design of novel anode materials with excellent performance and low cost can accelerate the commercialization of sodium-ion batteries [39,40,41,42,43,44,45,46,47,48]. Among the many anode electrode materials of sodium-ion batteries, hard carbon materials have the superiority of high capacity, low price, and low working voltage, and their unique structure is conducive to sodium-ion adsorption and reversible embedding/removal, showing excellent sodium storage performance, making them the most likely anode materials to be commercialized [49,50,51,52,53,54,55,56,57]. When commercializing hard carbon materials, troubles such as low first-cycle coulombic efficiency, terrible rate performance, and poor cycle stability are also faced [58,59,60]. In order to achieve an in-depth understanding of the sodium storage behavior of hard carbon anodes, this review analyzes in detail the mechanism of sodium-ion storage in hard carbon materials with different structures and then proposes three kinds of methods to improve the storage capacity of hard carbon materials, including morphology and structure construction, heteroatom doping, and electrolyte optimization. It is hoped that it can guide the synthesis of hard carbon materials with excellent properties and realize the application of sodium-ion batteries with hard carbon as the anode in large-scale national power grids as soon as possible.

## 2. Mechanism of Sodium Storage by Hard Carbon

**Figure 1:**
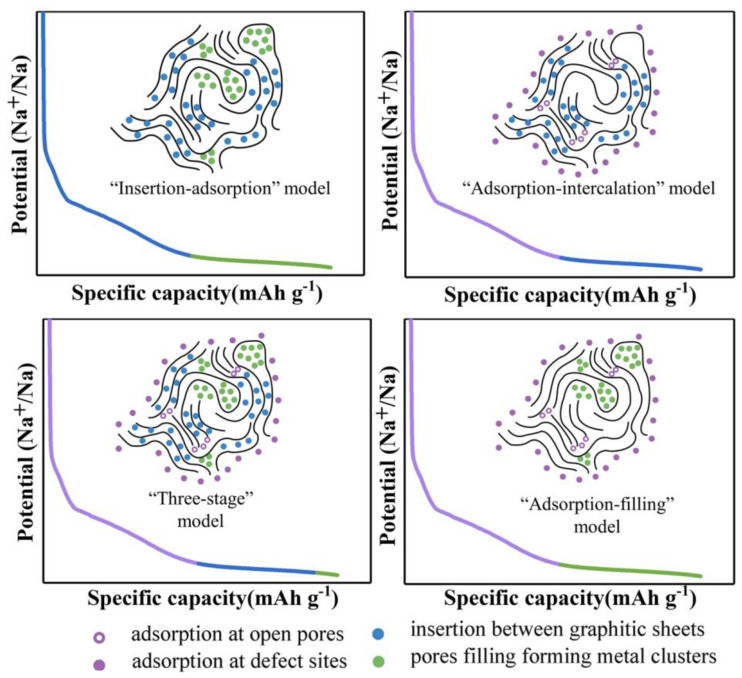
Four different mechanisms of sodium storage in hard carbon [77] (copyright Wiley, 2022).

Hard carbon is a kind of carbon material that cannot be graphitized at high temperatures, while graphite is a long-range ordered carbon material with a crystal structure [61,62,63,64,65,66,67,68]. The microstructure of hard carbon is obviously distinctive regarding graphite, which shows a short-range ordered structure with local graphite regions inside. Hard carbon prepared with various precursors has distinguishing characteristics, morphology, and structure, which makes the storage capacity of sodium ions vary greatly among hard carbon materials with distinguishable structures [69,70,71,72,73]. The difficulty in understanding the mechanism of sodium-ion storage in hard carbon has hindered the design of anode materials with excellent performance for sodium-ion batteries. Scientists have made many efforts to solve the controversial problems in developing sodium-ion batteries, and enormous achievements have been made during this period [74,75,76]. This review summarizes four different sodium-storage models for hard carbon anodes [77,78] (Figure 1), including the “embedding-adsorption” model [79,80], the “adsorption-embedding” model [3,81,82], the “three-stage model” [83], and the “adsorption-filling” model [84]. The following section describes the storage of sodium ions in hard carbon.

### 2.1. “Embedding-Adsorption” Model

**Figure 2:**
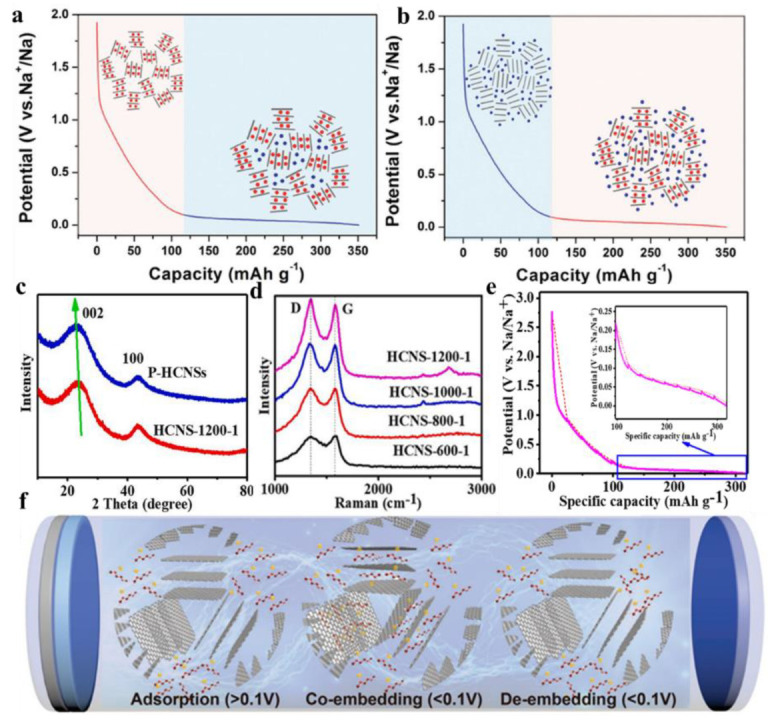
Model of sodium-storage mechanism using hard carbon; (**a**) embedding-adsorption mechanism; (**b**) adsorption-embedding mechanism [86] (copyright Wiley, 2017); (**c**) XRD of P-HCNS and CNS-1200-1; (**d**) Raman of HCNSs; (**e**) the GITT Curves of CNS-1200-1 [82] (copyright Elsevier, 2021); (**f**) schematic diagram of adsorption-co-embedding mechanism [91] (copyright Wiley, 2022).

This sodium storage model was first proposed by Stevens et al. in 2000. As a glucose precursor, hard carbon materials were synthesized by pyrolysis, which served as the anode of sodium-ion batteries [85]. The storage mechanism of sodium ions in a hard carbon negative electrode was proposed, with the sodium ions embedded into the carbon layer under a high-voltage range, then absorbed into the pores at a low-voltage range [86] (Figure 2a). Subsequently, Stevens et al. found that sodium ions could enter the layers of hard carbon materials through wide-angle in-situ X-ray diffraction studies, and the layer spacing expanded with the insertion process. They also conducted small-angle diffraction studies and found sodium ions in the nanopores of hard carbon materials [87]. Ilic et al. processed commercial hard carbon by ball milling at different lengths and studied its sodium ion storage mechanism through various characterization methods [79]. It was found that by extending the milling time, the amount of gas adsorption and the pore volume increased through physical nitrogen adsorption and small-angle X-ray diffraction.

On the basis of the quenched solid density functional theory model, the pore and specific surface area of the sample increases with an increase in ball milling time, confirming the presence of large quantities of closed pores in the sample. Electrochemical tests have confirmed that hard carbon materials with high closed porosity can store more charges in the platform region because of the existence of quasi-metallic sodium particles in the closed pores, which is consistent with the embedding-adsorption mechanism. Xu et al. used bamboo as a precursor and combined acidizing pretreatments and a two-step carbonization method to introduce the carbonyl group and closed micropores into bamboo-sourced hard carbon materials [88]. Through testing, it was found that the carbonyl group provided additional active sites, improving the reversible adsorption capacity of sodium ions at the slope areas, while the closed micropores promoted the storage capacity of the sodium ions in the platform range. There is an existing similar mechanism of sodium ion storage of the platform region in the embedding-adsorption model.

###  2.2.“Adsorption-Embedding” Model

The “embedding-adsorption” mechanism has been recognized by some people but has also been opposed by others. The core idea of the “embedding-adsorption” mechanism is that the capacity of the low-voltage platform region is positively correlated with the porosity of the anode material. By increasing the carbonization temperature, the sodium storage capacity of the low voltage platform region can be increased while the specific surface area of material is reduced, which is contrary to the “embedding-adsorption” mechanism. On this basis, the opposite mechanism of “adsorption-embedding” sodium storage was proposed [88] (Figure 2b). Cao et al. summarized the “adsorption-embedding” model in 2012. In the high-voltage region, sodium ions were adsorbed by the pores of the materials, while in the low-voltage range, they were inserted between the carbon layers [89]. Jin et al. used situ X-ray diffraction (situ XRD) to prove that the peak (002) was offset during the discharge-charge process [82] (Figure 2c). Figure 2d is the Raman image of the hard carbon near spheres (HCNSs). There are a couple of peaks at 1340 and 1580 cm^−1^ that indicate the existence of defects. By using the galvanostatic intermittent titration technique (GITT) test (Figure 2e), it was verified that the diffusion coefficient of sodium ions in the high-potential region is higher than in the low-voltage region. By using the *i = aν^b^* equation, it was proved that the diffusion process of sodium ions is dominant near 0.1 V, which further confirms the “adsorption-embedding” mechanism in hard carbon. Lu et al. synthesized hard carbon materials by a simple ball-milling method and confirmed the influence of the microstructure on the sodium storage capacity of hard anodes [90]. By increasing the milling time, the graphite-like nanoregions in hard carbon become smaller and thinner, and the specific surface area and micropore volume increased along with the degree of structural disorder, while the capacity and initial coulombic efficiency (ICE) of the platform region decreased. The capacity of the high-voltage region is derived from the activity and defect sites of the sodium ions adsorbed on the surface of the material. In contrast, the low-voltage region corresponds to the insertion of sodium ions into graphite layers, which is consistent with the “adsorption-embedding” mechanism.

Using asphalt as a precursor, Yuan et al. synthesized a battery using a carbon anode with diverse crystallinity and pore structures under various carbonization temperatures and finally proposed a universal sodium storage mechanism by combining it with electrochemical performance [92]. In a high-voltage region of greater than 0.3 V, the sodium ions adsorbed in the holes, defects, and impurity sites in the presence of the hard carbon. A low-voltage region of less than 0.3 V for the sodium ions is inserted into the middle of the carbon layer. Only hard carbon has a capacity in the plateau region of less than 0.1 V, resulting from the filling of sodium ions in closed pores. Jiang et al. proposed an “adsorption-coembedding” mechanism, that is, in a high-voltage region of greater than 0.1 V, the sodium ions adsorb in the amorphous region of hard carbon, and with the further progress of a discharge reaction, the sodium ions and ether-based solvent chelates co-embed in the platform region of less than 0.1 V [91] (Figure 2f). The “adsorption-embedding” mechanism is consistent with the Na^+^ storage mechanism of most hard carbon.

###  2.3.“Adsorption-Insertion-Hole Filling” Model

**Figure 3:**
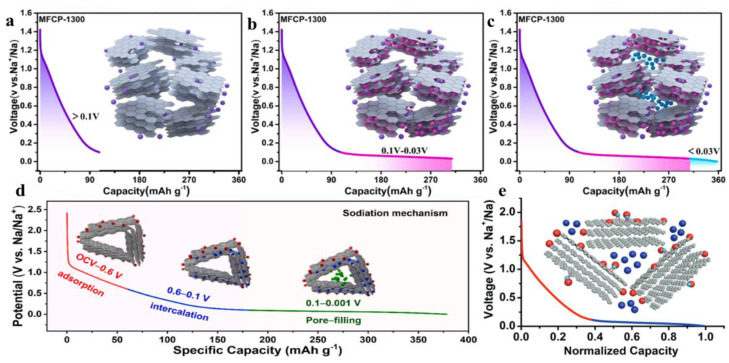
(**a**–**c**), schematic diagram of Na^+^ storage mechanism of MFCP-1300 [93] (**d**), schematic diagram of Na^+^ storage [94] (copyright Elsevier, 2022) (**e**), schematic diagram of adsorption-pore filling mechanism [95] (copyright Wiley, 2018).

Although there exist many experimental data from the two mechanisms of sodium storage demonstrated above, with the deepening of research, there are still many experimental phenomena that the above two mechanisms cannot explain. With the improvement of experimental methods and characterization techniques, researchers continue to discover new mechanisms of sodium-ion storage, such as the “adsorption-insertion-hole-filling” sodium storage mechanism. Ren et al. synthesized a series of independent, flexible microfiber carbon papers (MFCPs) as sodium-ion battery anode materials at a pyrolysis temperature of 900–1500 °C using filter paper as a precursor and a simple graphite plate-assisted method [93]. MFCP-1300 exhibited the most excellent electrochemical performance, including up to 96.3% ICE and a high platform capacity, attributed to a large graphite-like structure and low defect and porosity. The mechanism of sodium storage in MFCP-1300 can be divided into three parts: adsorption of sodium ions in edge and surface defects (>0.1 V), implantation of exposed sodium ions into the interlayer (0.1–0.03 V), and the filling of closed pores (<0.03 V) (Figure 3a–c). Song et al. used esterified starch as a carbon source to prepare hard carbon materials by quantitatively adjusting the oxygen content through low-temperature hydrogen reduction [94]. The storage mechanism of sodium ions in hard carbon materials was confirmed by cyclic voltammetry (CV) situ Raman (Figure 3d): In the first stage (open-circuit voltage (OCV) of 0.6 V), the sodium ions were adsorbed on the defect sites. Through low-temperature carbonization, the defect sites in hard carbon materials increase. The defects provide additional sodium-ion storage sites and improve the capacity retention rate at high rates. In the second stage (0.6–0.1 V), the sodium ions entered the graphite-like region. Thanks to a high proportion of graphite-like regions, hard carbon materials provide a suitable microstructure for sodium-ion insertion and high capacity in the inclined region. In the third stage (0.1–0.001 V), the sodium ions are filled in the closed micropore. When the insertion site was saturated, closed-hole filling was the main sodium storage behavior in the platform region.

###  2.4.“Adsorption-Hole Filling” Model

Bai et al. synthesized composite materials by injecting sulfur elements into hard carbon and confirmed the two-step storage behavior of sodium ions in anodes through experiments [95] (Figure 3e), showing the voltage curve change of hard carbon materials with a low carbonation temperature have higher defects and heteroatom concentration and are amorphous structures.

When increasing the carbonization temperature, the defects and heteroatom concentration in the materials can be reduced, and the graphite structure of materials can be improved. In the Na^+^ insertion process, Na^+^ adsorption at the defect or heteroatom site leads to a change in binding energy, which makes the voltage curve appear inclined. The pores of the hard carbon material were filled with Na^+^ and verified by a series of experiments, including the use of sulfur to fill the pores, varying the heating temperature to adjust the pore structure, the use of different electrolyte systems, the comparison of fuzzy and conflict spectral analyses in the literature, and the pores filled with Na^+^ with weak binding energy, similar to the deposit of sodium metal. The result is a platform-shaped low-pressure area. Fei et al. used cotton as a carbon source to pyrolyze hard carbon materials at 1300 °C [96]. Ex situ X-ray photoelectron spectroscopy (XPS) and GITT confirmed that the sodium storage mechanism of hard carbon materials was adsorption and pore filling. The inclined region above 0.12 V corresponds to the adsorption of Na^+^ between disordered graphite layers. Na^+^ fills the nanopore in the platform region near 0 V. Due to the different precursors and carbonization temperatures, hard carbon materials have a complex morphology and structure, and it is very difficult to determine the storage mechanism of sodium ions in them. In particular, the sodium storage mechanism in the platform area is controversial, which also puts forward clear requirements for future research.

## 3. Challenges and Solutions of Hard Carbon

### 3.1. Problems with Hard Carbon

As an anode of sodium-ion batteries, hard carbon has excellent performance, low cost, and uniform distribution, which is conducive to commercialization, but this also has the problem of a wasted rate performance and low ICE. Hard carbon materials have abundant internal pores and surface defects. The first charge and discharge process cause serious irreversible reactions, including electrolyte that is deposited at the electrode surface to form a solid electrolyte interphase (SEI) membrane, which consumes many sodium ions, surface defects, and internal pores in the cycle and will also cause irreversible reactions, which are the main reason for the low ICE of hard carbon materials. The capacity of the whole battery is influenced by active matter loss, so the specific surface area and defects of hard carbon can be reduced, and some pores can be closed to reduce the irreversible reaction to achieve the purpose of improving ICE and obtaining the anode materials of sodium-ion batteries.

### 3.2. Improvement Strategy

#### 3.2.1. Morphology and Structure Regulation

**Figure 4:**
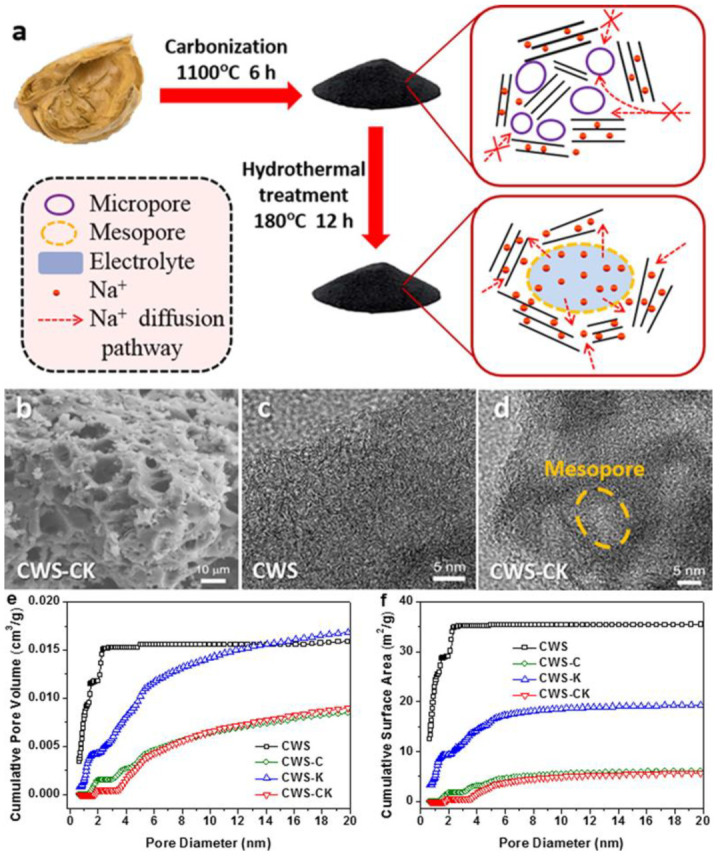
(**a**), Synthesis process of the hard carbon using walnut shell as a precursor. (**b**) SEM image of CWS-CK, (**c**,**d**) HRTEM images of CWS and CWS-CK, (**e**,**f**) specific pore volume and specific surface area of CWS-CK, CWS-C, CWS, and CWS-K [97] (copyright Elsevier 2020).

Yang et al. used walnut shell as a carbon source to prepare anode material through high-temperature carbonization and then hydrothermally treated hard carbon with cetyltrimethyl-ammonium bromide (CTAB)/KOH [97] (Figure 4a). Using a scanning electron microscope (SEM) to analyze CWS-CK (Figure 4b), it was shown that the surface presents a massive structure with micropores, which is conducive to electrolyte diffusion in the hard carbon main body. High-resolution transmission electron microscopy (HRTEM) images of CWS and CVS-CK can be seen in Figure 4c,d, respectively. Through comparison, it was found that the micropores of CVS-CK were transformed into mesoporous ones, which slow down the diffusion of sodium ions or even have difficulty containing Na^+^. On the contrary, mesoporous pores are beneficial to sodium-ion diffusion. The specific surface area and pore volume of the samples are plotted vs. the pore size, respectively (Figure 4 e,f). The pores of CWS concentrate at 0.6–2 nm. Besides, the mesopore volume of CWS-CK is larger than CWS. The CWS-CK contains abundant mesopores that accelerate Na^+^ intercalation. The maximum capacity of CWS-CK under 0.02 A g^−1^ can attain 283.7 mAh g^−1^, 83% higher than that of hard carbon that is rich in micropores, which still has a capacity of 189.4 mAh g^−1^ at 0.2 A g^−1^ after 320 cycles.

Jin et al. synthesized several spheroidal hard carbons under different carbonization conditions, and the effect of microstructure on Na^+^ storage behavior was investigated [82].

**Figure 5:**
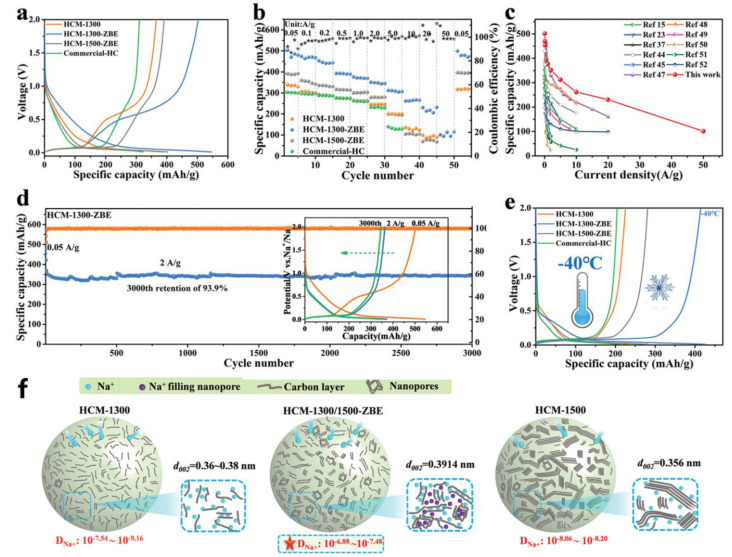
Electrochemical characterizations. (**a**) Galvanostatic charge/discharge (GCD) curves of HCM-1300, HCM-1300-ZBE, HCM-1500-ZBE, and commercial HC with the voltage range from 0.01 V to 2 V at 50 mAh g^−1^ of the second cycle. (**b**) Rate performances at 0.05, 0.1, 0.2, 0.5, 1.0, 2.0, 5.0, 10, 20, and 50 A g^−1^, respectively. (**c**) The electrochemical performance of different HC materials [15,23,37,44,45,47,48,49,50,51,52]. (**d**) The long-term cycling performance of HCM-1300-ZBE at 2 A g^−1^. (**e**) The second cycle of the GCD curve with −40 °C, (**f**) showing sodium-ion diffusion in HCM-1500, HCM-1300, and HCM-1300/1500-ZBE [98] (copyright Wiley 2022).

It was confirmed by experimental data that the overall sodium-ion storage capacity could be improved by increasing the material order and platform capacity while maintaining the appropriate layer spacing (>0.364 nm). The “adsorption capacity” of the high-voltage region could be markedly increased by increasing the specific surface area. The electrode material had a high capacity of 305 mAh g^−1^. The platform area had a capacity of 170 mAh g^−1^ and 210 mAh g^−1^ at 0.02 A g^−1^ and 1 A g^−1^, respectively. Yin et al. synthesized different hard carbon materials (HMM-1300-ZBE) through the ZnO auxiliary etching strategy, which can achieve extremely fast sodium-ion storage with a voltage between 0.01 V and 2 V, which increases the diffusion rate of sodium ions by two orders of magnitude [98]. Through characterization, it was proved that the short graphite layer could be made thicker and longer by increasing the oxygen content via a volume-etching strategy, which improves the ion transport rate. In the electrochemistry test, the discharge capacity of HCM-1300-ZBE at the first cycle is 501 mAh g^−1^ at 0.05 A g^−1^ (Figure 5a), and HCM-1300-ZBE shows excellent rate performance. The capacity is 230.4 mAh g^−1^ and 107 mAh g^−1^ at 20 A g^−1^ and 50 A g^−1^ (Figure 5b,c), and the outstanding cycle stability is 344 mAh g^−1^ at a current density of 2 A g^−1^. The capacity retention rate is more than 99.99% after 3000 cycles (Figure 5d). The electrodes were used at a wide range of temperatures, with a capacity of 426 mAh g^−1^ under −40 °C, a mere 14.9% reduction from 25 °C, which is the lowest reported in the literature. Figure 5f shows the diffusion and storage of sodium ions of HCM-1500/1300 and HCM1300/1500-ZBE. Finally, the whole battery was tested using the negative electrode of HCM-1300-ZBE and the positive electrode of Na_3_V_2_(PO_4_)_3_(NVP). It was found that the capacity reached 39.95 mAh g^−1^, and the average voltage of the whole battery reached 3.31 V at 10 A g^−1^. The power densities and energy densities are 6.73 kW kg^−1^ and 294.6 Wh kg^−1^, respectively.

Liu et al. synthesized many permeable channel-like hard carbons with interconnections through an ingenious controlled phase-transition method, improving ionic diffusion and electrode and electrolyte interface and affinity [99]. The experimental results show that porous hard carbon materials with a crosslinked mesoporous structure can improve the capacity of sodium-ion storage. When compared with other initial hard carbon materials with lower pore content, the ICE of the synthesized porous hard carbon anode increased from 51.5% to 68.3%, with a high capacity of 332.7 mAh g^−1^ at 50 mA g^−1^, as well as a capacity retention rate enhanced from 46.5% to 67.4% at 2 A g^−1^. The capacity retention rate was enhanced from 86.4% to 95% after 90 cycles, with anode capacity, cycle stability, and rate performance all comprehensively enhanced. Yang et al. used melt diffusion carbonization to make the micropores inside of hard carbon become ultrafine pores [100]. In situ XRD confirmed that the ultrafine pores provided additional sodium-ion storage sites, increasing the capacity of the anode. The ICE of hard carbon increased up to 80.6%, with a capacity of 346 mAh g^−1^ at 0.03 A g^−1^, and regions with a voltage of less than 1 V contributed more than 90% to the capacity. The surface capacity was 5.32/6.14 mAh cm^−2^ at −20 °C and 25 °C, respectively, under a high load of 19 mg cm^−2^. Porous hard carbon with a 3D structure can be constructed by morphological and structural regulation to realize the fast diffusion of sodium ions and improve the cycle stability and rate performance of sodium-ion batteries.

#### 3.2.2. Doping by Heteroatom

**Figure 6:**
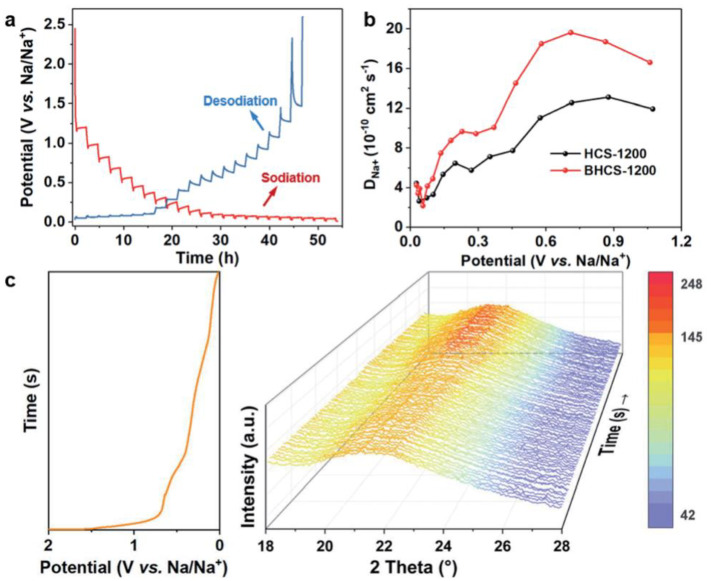
(**a**) GITT curve of BHCS-1200. (**b**) D_Na+_ of BHCS-1200 and HCS-1200. (**c**) Operando XRD patterns of BHCS-1200 at 30 mAh g^−1^ [101] (copyright The Royal Society of Chemistry 2022).

The number of defects and layer spacings can be regulated by heteroatom doping. Wu et al. adopted a simple boron-doping strategy to optimize the multiscale carbon-based structure in terms of spherical morphology and crystal parameters while improving the storage capacity of the sodium ions and the battery’s electrochemical performance [101]. By using boric acid as a boron source and glucose as a precursor, hard carbon nanospheres were synthesized through high-temperature carbonization, presenting a polydisperse nanosphere shape and expanded layer spacing. These structural features can improve sodium-ion storage and rate performance. The capacity of the hard carbon anode in the full voltage range significantly increased under boron doping, with the capacity of the high-voltage region increased by three times, while the low-voltage region increased by 67%. The reaction curve of the B-doped hard carbon spheres (BHCS-1200) negative electrode at a pulse current of 0.03 A g^−1^, with the calculated chemical diffusion rate of the sodium ions (D_Na+_), was calculated (Figure 6a). In Figure 6b, the D_Na+_ of the two electrodes is compared, and it is found that the diffusion mechanism of Na^+^ in the two electrode materials has many similarities, indicating that doping boron atoms do not change the mechanism of sodium storage behavior. From 0.1 to 0.03 V, the D_Na+_ of BHCS-1200 was higher than HCS-1200, illustrating that heteroatom doping increased the storage capacity of the sodium ions. The capacity of the region with a voltage larger than 0.1 V comes from the sodium-ion filling of the micropores. The in-situ XRD pattern of the BHCS-1200 electrode at 30 mA g^−1^ can be seen in Figure 6c. At 21°~22°, BHCS-1200 has a strong peak, indicating that the layer spacing of BHCS-1200 becomes wider after the initial discharge process, confirming that sodium-ion embedding occurs in the low discharge region. The density functional theory (DFT) calculation of BHCS-1200 confirmed that the diffusion and intercalation kinetics of the sodium ions in doped hard carbon improved, and the intercalation capacity increased. Yu et al. used tamarind fruit as a carbon source to prepare hard carbon materials through a cost-effective and scalable self-assembly technology through the natural super accumulation and enrichment of calcium ions [102]. After further hydrothermal treatment, self-binding and evenly distributed calcium ions act as a buffer layer to expand the distance between hard carbon layers. By optimizing the calcining temperature, the natural pore structure can be retained to a large extent. The hard carbon anode rich in calcium ions shows excellent performance. The capacity was 326.7 mAh g^−1^ after 250 cycles at 0.05 A g^−1^, and the ICE was 70.39%.

**Figure 7:**
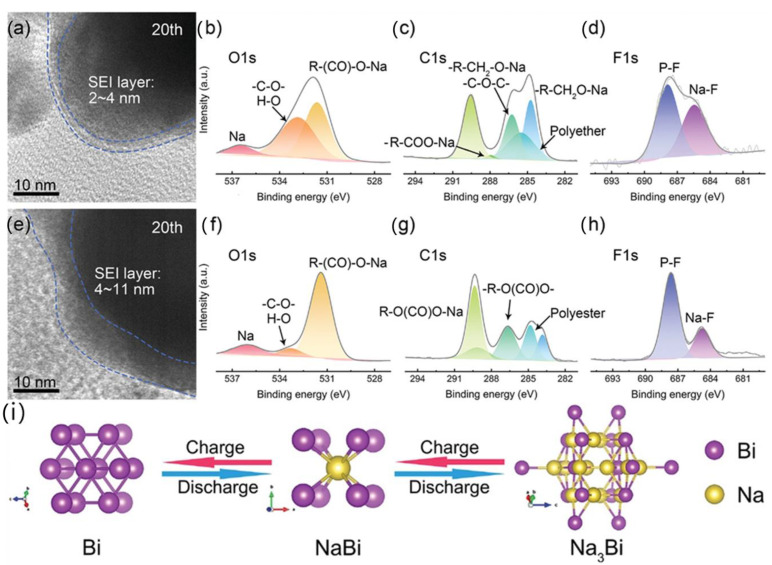
(**a**) HRTEM images of the Bi@C⊂CFs after 20 cycles in DME-based (**a**) and EC/DEC-based (**e**) electrolytes. (**b**–**d**,**f**–**h**) The high-resolution XPS spectra of C, O, and F for Bi@C⊂CFs with EC/DEC-based and DME-based electrolytes after cycles. (**i**) Reaction mechanism of Bi in charge and discharge process [103] (copyright Wiley 2022).

Liang et al. proved that the carbon nanoribbon array modified by metallic bismuth nanospheres coated with a carbon layer was synthesized by introducing MOF, which had a three-dimensional frame superstructure [103]. It was found that a thin and uniform SEI film was generated on the anode surface during the discharge and charging cycles, and the porous structure formed on the electrode surface not only improved the utilization rate of active substances but also reduced the diffusion distance of electrons or ions. As shown in Figure 7, they analyzed the thickness and chemical composition of the SEI film carbon nanobelt arrays decorated with carbon-layer-coated metallic Bi nanospheres (Bi@C⊂CFs) in the two matrix electrolytes of dimethyl ether-based (DME) and ethylene carbonate (EC)/diethyl carbonate (DEC)-based by ex-in situ HRTEM and XPS. Figure 7a,e show HRTEM images of the anodes after 20 cycles in the DME-based and EC/DEC-based electrolytes, respectively. In the DME-based electrolyte, the SEI film is homogeneous, and the main component is RCH_2_ONa (Figure 7b–d). In the EC/DEC-based electrolyte, the SEI film is thick and uneven, and the main component is ROCO_2_Na, which easily decomposes, resulting in the increased thickness of the SEI film (Figure 7f–h). The alloying/dealloying models of Bi in a sodium-ion battery are shown in Figure 7i. The Bi@C⊂CFs material shows excellent electrochemical properties, an outstanding performance of 305 mAh g^−1^ at 5 A g^−1^ past 5000 cycles, an outstanding capacity of 308.8 mAh g^−1^ at 80 A g^−1^; good cycle performance provides the possibility for commercial applications.

**Figure 8:**
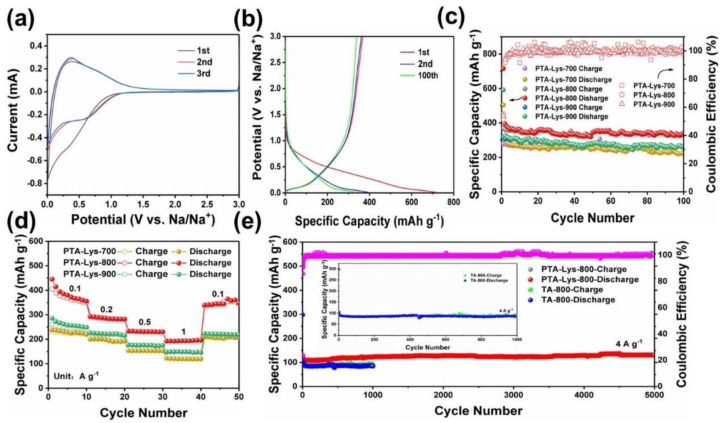
(**a**), CV curve of PTA-Lys-800. (**b**) The GCD curve of PTA-Lys-800 at 100 mA g^−1^. (**c**) Electrochemical stability of PTA-Lys-800 at 100 mAh g^−1^. (**d**) Rate performance of PTA-NHCS-T. (**e**) Cycling performance of PTA-Lys-800 and TA-800 at 4 A g^−1^ [107] (copyright Wiley 2022).

Wang et al. synthesized phosphorus-doped hollow carbon nanorods (P-HCNs) with a phosphorus content of 7.5% via a one-step method [104]. The P-HCNs showed a high capacity of 260 mAh g^−1^ at 1.0 A g^−1^ after 500 cycles and high ICE (73%). The mechanism of different contents of phosphorus on the structure of hard carbon was investigated by XRD, XPS, and Raman spectrum techniques. Li et al. obtained several phosphorus-doped hard carbon materials by directly calcinating phospho-solidified epoxy resin under various temperatures. They analyzed the evolution of the microstructure and groups containing phosphorus as well as the sodium-storage properties with increases in temperature [105]. By raising the carbonization temperature, the phosphorus groups of P-O and P-C become P-P bonds with higher activity, and the layer spacing presents a non-monotone upward trend. The increase in active phosphorus groups and the increase in layer spacing are conducive to providing more sodium-ion transport channels and active sites. With a high capacity of 379.3 mAh g^−1^ at 100 mAh g^−1^, and the outstanding rate performance was as high as 158.1 mAh g^−1^, with long cycle stability over 6500 cycles at 5.0 A g^−1^ and nearly no capacity loss. Yan et al. used ordinary PCl_3_ and C_6_H_12_ as the phosphorus and carbon sources to successfully establish a nitrogen-bulking anaerobic reaction system and simultaneously realized carbonization and in-situ P doping [106]. In the synthesized hard carbon material, the mass fraction of phosphorus reached 30%, which was higher than previously reported phosphor-doped carbon material, and the phosphorus atoms replaced the carbon atoms embedded in the lattice. The adsorption energy of the sodium ions and intercalation spacing increased significantly. There was a reversible capacity of 510.4 mAh g^−1^ with a rate performance of 397.1 mAh g^−1^ at 10 A g^−1^ at a working voltage of 0.54 V. Huang et al. used tannic acid (TA) and amino acid as a carbon source to synthesize a high proportion of active nitrogen-doped hard carbon (PTA-Lys-800) [107]. The electrochemical performance of the PTA-Lys-T//Na cell is shown in Figure 8. The first 3 cycles of the CV curve shown in Figure 8a is the PTA-Lys-800 at 0.2 mV s^−1^. Under a scan speed of 0.2 mV s^−1^ for the first three cycles**,** the CV curve of PTA-Lys-800 can be seen in Figure 8a. A region of irreversible regions appears between 0.01 V and 0.65 V due to the formation of the SEI film. In the subsequent cycles, the curves almost coincide, showing that PTA-Lys-80 has excellent reversibility.

Figure 8b shows the GCD curves of the sodium-ion batteries when the voltage is 0.01–3.0 V. It can deliver 318.3, 504.0, and 715.4 mAh g^−1^ at initial discharge, while the initial charge capacities are 591.1, 504.0, and 715.4 mAh g^−1^ for PTA-Lys-900, PTA-Lys-800, and PTA-Lys-700, respectively. The ICE is 55.2, 51.3, and 53.8%. The PTA-Lys-800 delivered excellent cycling performance, with a high reversible capacity of 338.8 mAh g^−1^ at 100 mA g^−1^ after 100 cycles and a capacity retention rate of over 85% (Figure 8c). The PTA-Lys-800 electrode delivered an outstanding rate performance (Figure 8d). The PTA-Lys-800 electrode showed a high reversible capacity of 131.1 mAh g^−1^ after 5000 cycles at 4 A g^−1^, demonstrating that the long-term cycling stability of PTA-Lys-800 is better than the TA-800 electrode (Figure 8e). The number of active sites can be increased by simple heteroatom doping to enhance the storage capacity of sodium ions. The sodium-ion battery anode materials designed by single-atom doping have high capacity and excellent cyclic stability, but further studies are needed to understand the mechanism more deeply.

#### 3.2.3. Electrolyte Optimization

Niitani et al. confirmed that all-solid sodium-ion batteries with a hard carbon anode and a sodium carborane electrolyte (Na (CB_9_H_10_)_0.7_(CB_11_H_12_)_0.3_) have excellent fast-charging performance and verified that (Na (CB_9_H_10_)_0.7_(CB_11_H_12_)_0.3_) has good plasticity through experiments [8]. By generating a thin oxide layer to establish a tight, stable connection between the electrolyte and electrode interface, reducing the interface impedance of the active material load of hard carbon to 5.8 mg cm^−2^ (0.68 Ω cm^−2^ at 0.1 V vs. Na^+^/Na), the full battery has an area capacity over 1 mAh cm^−2^ for 5 min. Yu et al. designed a homotype heterojunction using hard carbon to generate a steady, solid electrolyte interface, effectively increasing the ICE by 16.4% [58]. A simple surface engineering strategy was used for the construction of a homogeneous amorphous Al_2_O_3_ layer using HC, which shielded the active site. Finally, electrolyte decomposition and adverse factors were slowed down by inhibiting the decomposition of NaPF_6_ in ether-based electrolytes and reducing the accumulation of NaF, forming a thin and dense solid electrolyte interface to reduce interface impedance, improving not only the ICE but also the storage capacity of sodium ions. The optimized reversible capacity of the hard carbon anode occurred at 321.5 mAh g^−1^ at 50 mA g^−1^. After 2000 cycles at 1 A g^−1^, the capacity retention rates of the optimized hard carbon and untreated hard carbon were 86.9% and 52.6%, respectively. Ma et al. used hard carbon to study the relationship between SEI films and sodium-ion storage performance and found that a good SEI film is not directly related to its components, such as NaF and Na_2_O [108]. However, the fine structure arrangement of the components of a nano SEI film is the basis for achieving fast sodium-ion storage and a stable interface for a “good” SEI film. A double-layer SEI film composed of the inner layer of inorganic matter and the outer layer of organic matter has a long cycle life and outstanding rate performance. The SEI membrane is the gateway for sodium ions from the electrolyte to the electrode. In order to transform the crystal structure of the hard carbon surface using a cylindrical solvent and play the role of “pseudo-SEI”, a 1 M NaPF_6_^−^ inTEGDME electrolyte is used, which also facilitates the fast and stable storage of sodium ions, with an excellent performance of 192 mAh g^−1^ at 2 C and a cycle stability of 1100 cycles at 0.5 C. The interface stability of hard carbon materials over long-term cycles is poor. The continuous accumulation of electrolyte decomposition substances will seriously increase the interface impedance and sharply decrease the discharge capacity. Zhang et al. used n-phenyl bis (trifluoridemethansulfonimide) as an electrolyte film-forming additive, and could effectively improve the long-cycle performance of the hard carbon negative electrode of sodium-ion batteries, making the cyclic stability of half-batteries increase from 0% to 50% after 500 cycles, and the improved interphase stability makes the capacity retention rate of the whole battery increase by 52% after 100 cycles [109]. Jin et al. designed a non-flammable local high concentration electrolyte for highly reversible sodium-ion batteries [110]. Through high-resolution cryo-TEM, it was found that an extremely thin but solid interface layer was formed on the positive electrode surface, which could inhibit the transition metal dissolution and reduce the structural changes on the electrode surface. The formation of a solid electrolyte interface rich in inorganic substances on the hard carbon surface can reduce the side reactions between hard carbon and an electrolyte. These stable interfaces ensure the high coulombic efficiency and long-cycle stability of the hard carbon negative electrode, with a capacity of 247.9 mAh g^−1^ after 500 cycles and a capacity retention rate of 94.8%. Lohani et al. formed a thin, dense SEI film on the surface of hard carbon by directly contacting hard carbon that was moistened with a vinyl carbonate electrolyte with sodium metal [111]. During incubation, the hard carbon negative electrode was partially passivated by a thin SEI film. This sodium-ion cell had a first-cycle coulombic efficiency of 97% and provided a stable area capacity of 1.4 mAh cm^−2^ at 0.1 mA cm^−2^ at a current rate of 0.1 mA cm^−2^, showing excellent rate capacity. With a high current density of 0.5 mA cm^−2^, the initial capacity of 1.18 mAh cm^−2^ was maintained, and excellent cycling stability was achieved with 1.0 mAh cm^−2^ after 500 cycles. Dong [112] found that the sodium storage performance of a hard carbon (HC) anode in ether electrolytes is higher and more effective than conventional ester electrolytes. The experiment results confirm that the resistances originated from the charge transfer (Rct) and SEI film (RSEI) in EC/DEC-based electrolytes are both higher than those in DEGDME-based electrolytes, that is, 137.1 Ω versus 64.36 Ω for Rct and 111.6 Ω versus 23.93 Ω for RSEI, respectively. Therefore, these results imply the facilitation of Na storage kinetics in the DEGDME-based electrolyte. Zeng et al. constructed a discontinuous SEI film on a hard carbon negative electrode by regulating NaPF6 in an ether-based electrolyte according to the storage mechanism of sodium ions in the low-pressure range, including the co-embedding of sodium ions into the carbon layer and the desolating sodium ions into the hole of the carbon [113]. The discharge capacity was 459.7 mAh g^−1^ at 0.1 C, and the capacity was 357.2 mAh g^−1^ at 1 C after 500 cycles, with both being higher than other electrolytes. Hou et al. directly demonstrated the internal relationship between solvent structure and electrochemical reaction by controlling the anions in the solvent shell, which determined the storage kinetics of the sodium ions and the SEI membrane’s evolution process [114]. The results showed that the weak coulomb interaction between Na^+^ and PF_6_^−^ could improve the transport volume and storage kinetics of Na^+^ in liquid. PF_6_^−^ is induced to preferentially reduce and promote the addition of the additive into the solvated structure to form a thin, compact, protective, and layered form of SEI. Excellent SEI can effectively protect the anode, inhibit solvent decomposition, and improve battery performance. As expected, the 5 Ah Na_3_V_2_(PO_4_)_3_ hard carbon pouch cells with a well-matched PF_6_^−^-based electrolyte showed a high specific energy of 129 Wh kg^−1^, a superior capacity retention rate of 90% at 5 C after 700 cycles. The electrolyte plays an important role in the transfer of sodium ions between the anode and cathode. A thin and dense SEI film can be formed via an ether-based electrolyte, which can enhance the rate capacity of a sodium-ion battery with mixed ester-based electrolytes, although efforts have been made recently to develop advanced electrodes and electrolytes. Simultaneously, one of the key components of sodium-ion batteries is the separator [115].

**Figure 9:**
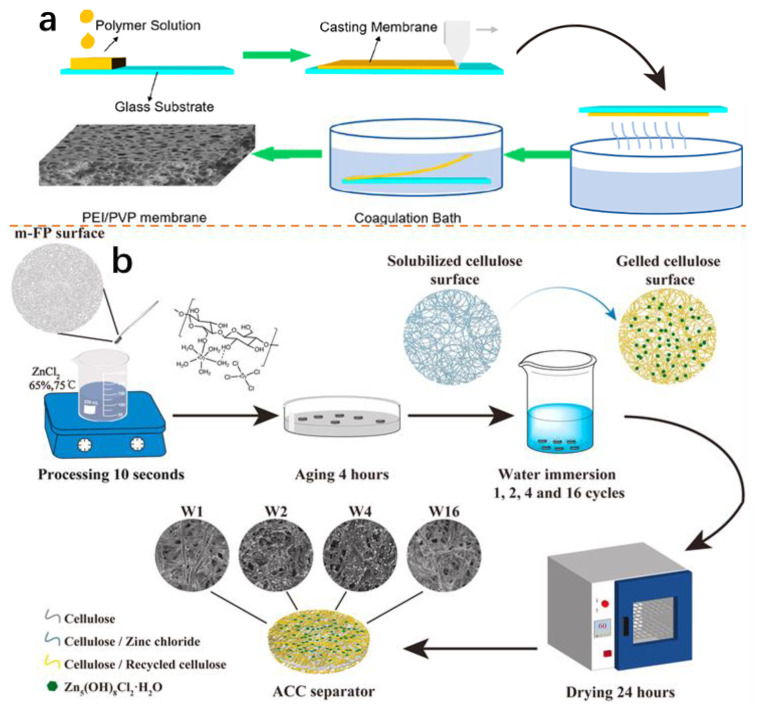
(**a**), Preparation process of PEI/PVP separators (Copyright, American Chemical Society 2021). (**b**) Preparation process of ACC separators (Copyright, Elsevier 2021).

Kang et al. induced a thin and compact SEI by a functionalized separator with an sp^2^ carbon conjugated-covalent organic framework (sp^2^ c-COF). A NaF-rich SEI can be generated by using a COF separator to destroy the structure of NaPF_6_ in the electrolyte. With a current density of 20 mA cm^−2^, the Na|COF|Na symmetric cell exhibits stable cycling stability over 1500 h, and the cell presented excellent rate performance (50C) and outstanding cycling stability over 5000 cycles at 10 C [116]. Niu et al. prepared a separator using an ether imide (PEI) or polyvinylpyrrolidone (PVP) mixture by immersion phase separation (Figure 9a). The PEI/PVP separator exhibited high ionic conductivity (1.14 mS cm^−1^), excellent thermal stability (over 180 °C), and outstanding flexibility and mechanical strength. The carbon/Na cell showed a high reversible capacity of 119.4 mAh g^−1^ at 0.5 C and good cycling performance [117]. Zhou et al. prepared all-cellulose composite (ACC) separators coated with Zn_5_(OH)_8_Cl_2_⋅H_2_O (ZHC) particles (Figure 9b). Batteries benefit from ZHC on the surface because it promotes sodium-ion passage and improves cycling performance. The hard carbon/Na cell showed outstanding cycling stability and a high reversible specific capacity of 315 mAh g^−1^ at 0.025 A g^−1^ [118]. Separators are part of sodium-ion batteries and are proposed to allow for the fast diffusion of sodium ions.

Table 1 shows some of the recent keywords related to hard carbon as an anode in sodium-ion batteries.

## 4. Summary and Outlook

Hard carbon, as a material for an anode in a sodium-ion battery, has the characteristics of high capacity, low cost, and wide distribution, showing great commercial value. Using hard carbon as the material for the anode of sodium-ion batteries has excellent application prospects in large-scale energy storage systems. Hard carbon has been commercialized, which has the advantage of low cost, but also has the problem of low capacity. In order to increase the value of hard carbon in practical applications, the next goal is to enhance the capacity of hard carbon. This paper first introduces four different mechanisms of sodium storage using hard carbon, including the “embedding-adsorption”, “adsorption-embedding”, “adsorption-insertion-pore filling”, and “adsorption-pore filling” cashier models. Based on sodium storage mechanism analysis, the problems of poor cycling performance and first-cycle coulombic efficiency regarding hard carbon materials were put forward, and three optimization strategies, including structural morphology regulation, heteroatom doping, and electrolyte regulation, were introduced to solve these problems. The hard carbon anode materials were analyzed and summarized below.

There are different ways to store sodium when synthesizing hard carbon materials using different precursors, and the mechanism of storing sodium in the low-voltage region is controversial. The development of advanced characterization techniques is crucial for clarifying the storage mechanism of sodium ions in the platform region, and the detailed storage behavior of sodium can guide the design of hard carbon negative electrodes.

The number of surface defects can be increased by heteroatomic doping, changing the conductivity of the electrode materials, and expanding the layer spacing of graphite, which improves the adsorption capacity of sodium ions, but also improves the diffusion rate, and finally achieves improvements in its capacity and rate performance. Adjusting the morphology, such as increasing the proportion of closed micropores and reducing the specific surface area of hard carbon, can reduce the consumption of sodium ions and improve the capacity and ICE during the production of SEI film.

An excellent electrolyte can form a dense and thin SEI film on the anode surface to reduce the impedance of sodium ion diffusion. Simultaneously, the introduction of additives to reduce side reactions and electrolyte decomposition can improve the stability of the electrolyte and elevate the cycle stability and rate performance of sodium-ion batteries. Developing biomass-based precursors that are low-cost and abundant resources and synthesizing hard carbon with a lower defect degree and a small specific surface area are both conducive to commercializing sodium-ion batteries.

## Figures and Tables

**Table 1:** Summary of representative publications on hard carbon as a SIB anode.

Precursor	Temperature (°C)	Electrolyte System	Doped Heteroatom (1)/Regular(2)	Current Density (mA g^−1^)	Capacity (mAh g^−1^)	ICE (%)	Cycle Number	Capacity Retention Rate (%)	Ref
hazelnut shell	1400	1 M NaPF_6_ in EC/DMC	2	20	342.0	91	100	91	[119]
fungus-treated basswood	1300	1 M NaPF_6_ in DEDM	2	200	175.6	88.2	536	86.4	[120]
sucrose	1300	1 M NaPF_6_ DEGDME	2	30	282.6	91.2	100	92	[121]
H_2_C_2_O_4_·2H_2_O	1300	1 M NaClO_4_ PC with 5% FEC	1	100	278.0	68.7	100	93.8	[49]
Epoxy phenol novolac resin	1800	1 M NaPF_6_ in DME	2	50 500	480.0 423.2	84.6	1000	92	[122]
trisodium citrate/hexamethylenetetramine	800	1 M NaClO_4_ PC with 5% FEC	2	5000	238.0	84.5	5000	98. 7	[123]
sugarcane waste-derived	1200	1 M NaClO_4_ PC with 5% FEC	2	50	323.6		500	96	[110]
sucrose	1200	1 M NaPF_6_ in EC/DEC	2	50 1000	318.0 77.90	97.1	2000	86.9	[58]
C_6_H_12_O_6_⋅H_2_O	600	1 M NaPF_6_ in EC/DMC		120 1200	109.0 98.2		300 600	80.3 71.8	[124]
Hard carbon		1 M NaPF_6_ in EC/DEC	2	400	153.2	84.49	300	94.8	[125]
citrate sodium	800	1 M NaPF_6_ in EC/DEC with 5%FEC	1	50 1000	280 193		200 2000	99 115	[126]
(H3BTC)	600	1 M NaPF_6_ in EC/DEC	1	5000	305		5000	89.4	[103]
sodium ligninsulfonate	1200	1 M NaPF_6_ in DME	2	20	210		1000	99	[82]
water-soluble starch	900	1 M NaClO_4_ in EC/DEC	2	25 100	343.1 197.8	80.1	200	87.2	[127]
3-aminophenol	1300	1 M NaPF_6_ in DEDM	2	2000	344		3000	99.9	[50]
corn starch	1100	1 M NaClO_4_ in EC/DEC	2	50	270		50	85.2	[124]

## Data Availability

All date that support the findings of this study are include within the article.
